# DNA barcode data accurately assign higher spider taxa

**DOI:** 10.7717/peerj.2201

**Published:** 2016-07-20

**Authors:** Jonathan A. Coddington, Ingi Agnarsson, Ren-Chung Cheng, Klemen Čandek, Amy Driskell, Holger Frick, Matjaž Gregorič, Rok Kostanjšek, Christian Kropf, Matthew Kweskin, Tjaša Lokovšek, Miha Pipan, Nina Vidergar, Matjaž Kuntner

**Affiliations:** 1 National Museum of Natural History, Smithsonian Institution, Washington, D.C., United States; 2 Department of Biology, University of Vermont, Burlington, Vermont, United States; 3 EZ Lab, Institute of Biology, Research Centre of the Slovenian Academy of Sciences and Arts, Ljubljana, Slovenia; 4 Department of Invertebrates, Natural History Museum Bern, Bern, Switzerland; 5 Department of Biology, Biotechnical Faculty, University of Ljubljana, Ljubljana, Slovenia; 6 Department of Biochemistry, University of Cambridge, Cambridge, United Kingdom

**Keywords:** Taxonomic impediment, Family, Genus, Global Genome Initiative, Genome, DNA barcoding

## Abstract

The use of unique DNA sequences as a method for taxonomic identification is no longer fundamentally controversial, even though debate continues on the best markers, methods, and technology to use. Although both existing databanks such as GenBank and BOLD, as well as reference taxonomies, are imperfect, in best case scenarios “barcodes” (whether single or multiple, organelle or nuclear, loci) clearly are an increasingly fast and inexpensive method of identification, especially as compared to manual identification of unknowns by increasingly rare expert taxonomists. Because most species on Earth are undescribed, a complete reference database at the species level is impractical in the near term. The question therefore arises whether unidentified species can, using DNA barcodes, be accurately assigned to more inclusive groups such as genera and families—taxonomic ranks of putatively monophyletic groups for which the global inventory is more complete and stable. We used a carefully chosen test library of CO1 sequences from 49 families, 313 genera, and 816 species of spiders to assess the accuracy of genus and family-level assignment. We used BLAST queries of each sequence against the entire library and got the top ten hits. The percent sequence identity was reported from these hits (PIdent, range 75–100%). Accurate assignment of higher taxa (PIdent above which errors totaled less than 5%) occurred for genera at PIdent values >95 and families at PIdent values ≥ 91, suggesting these as heuristic thresholds for accurate generic and familial identifications in spiders. Accuracy of identification increases with numbers of species/genus and genera/family in the library; above five genera per family and fifteen species per genus all higher taxon assignments were correct. We propose that using percent sequence identity between conventional barcode sequences may be a feasible and reasonably accurate method to identify animals to family/genus. However, the quality of the underlying database impacts accuracy of results; many outliers in our dataset could be attributed to taxonomic and/or sequencing errors in BOLD and GenBank. It seems that an accurate and complete reference library of families and genera of life *could* provide accurate higher level taxonomic identifications cheaply and accessibly, within years rather than decades.

## Introduction

Accurate identification of biological specimens has always limited the application of biological data to important societal problems. Obstacles are well-known and difficult: the vast majority of species are undescribed scientifically (Erwin, 1982; May, 1992; Mora et al., 2011); some unknown but large fraction of higher taxa are not monophyletic (Goloboff et al., 2009; Pyron & Wiens, 2011); many species can only be identified if certain life stages are available, e.g., adults (Coddington & Levi, 1991), classical data sources such as morphology imperfectly track species identity; the discipline of taxonomy continues to dwindle (Agnarsson & Kuntner, 2007); the classical process of taxonomic identification is mostly manual and cannot scale to provide the amounts of data required for real-time decisions such as environmental monitoring, invasive species, climate change, etc.

DNA sequence data potentially can eliminate most of these obstacles. DNA barcoding uses a fragment of the mitochondrial gene cytochrome *c* oxidase subunit I (CO1) as a unique species diagnosis/identification tool in the animal kingdom (Hebert et al., 2003), with analogous single to several locus protocols applied for vascular plants, ferns, mosses, algae and fungi (Saunders, 2005; Kress & Erickson, 2007; Nitta, 2008; Chase & Fay, 2009; Liu et al., 2010), protists (Scicluna, Tawari & Clark, 2006), and prokaryotes (Barraclough et al., 2009). Due to relative ease and inexpensive sequencing, DNA barcoding is a popular tool in species identification and taxonomic applications (e.g., Doña et al., 2015; Xu et al., 2015; see also Collins & Cruickshank, 2013), and the method is no longer fundamentally controversial at the species level (Pentinsaari, Hebert & Mutanen, 2014; Lopardo & Uhl, 2014; Čandek & Kuntner, 2015; Anslan & Tedersoo, 2015; Wang et al., 2015).

While most species remain undescribed, the situation is not so dire for larger monophyletic groups such as clades accorded the Linnaean ranks of genus or family. In assessing the state of knowledge about biodiversity, it is important to distinguish between the first scientific discovery of an exemplar of a lineage, and phylogenetic understanding of that lineage. Phylogenetic understanding—both tree topology and consequent taxonomic changes, are research programs with no clear end in sight. Linnaean rank is partially arbitrary, and one expects that the number of higher taxa will probably increase over time as understanding improves. Discovery, however, can have an objective definition: the year of the earliest formal taxonomic description of a member of the lineage or taxonomic group in which it is currently included. By this definition the earliest possible discovery of an animal lineage is 1758 (Linnaeus, 1758), or in the case of spiders, 1757 (Clerck, 1757).

**Figure 1:**
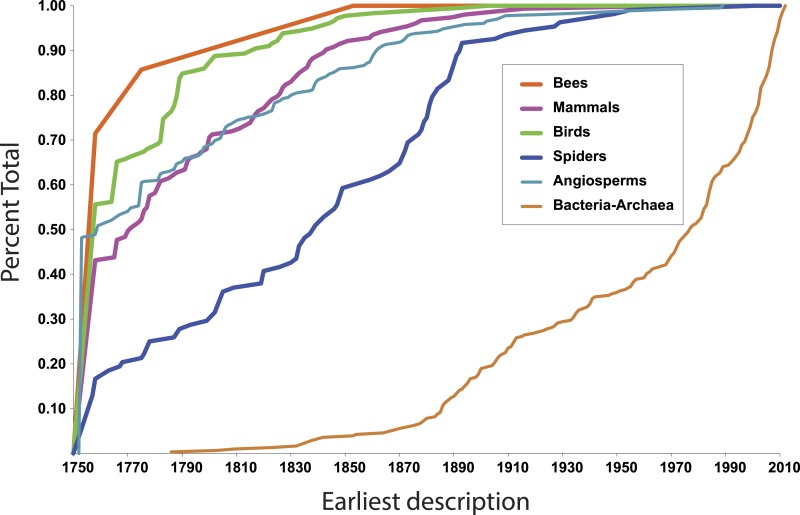
First discovery of major clades of life. Accumulation curve of dates of first discovery (year of first description of a contained species) of families for six major clades of life, 1758–2010.

More illuminating are the latest discoveries of lineages with the rank of family within larger clades, because the data tell us something about progress towards broad scale knowledge of biodiversity. The species representing the most recent discovery of a family of birds, for example, is the Broad-billed Sapayoa, *Sapayoa aenigma* Hunt, 1903 (Sapayoaidae). The species representing the most recently discovered mammal family is Kitti’s hog-nosed bat, *Craseonycteris thonglongyai* Hill, 1974 (Craseonycteridae). For flowering plants, it is *Gomortega keule* (Molina) Baill, 1972 (Gomertegaceae). For bees, it is *Stenotritus elegans* Smith, 1853 (Stenotritidae). For spiders, a megadiverse and poorly known group, it is *Trogloraptor marchingtoni* Griswold, Audisio & Ledford, 2012 (Trogloraptoridae), but the second most recent discovery of an unambiguously new spider family was in 1955, Gradungulidae (Forster, 1955). Figure 1 illustrates the tempo of first discovery of families for these five well-known clades. At the family level, these curves are essentially asymptotic, implying that science is close to completing the inventory of clades ranked as families for these large lineages. On the other hand, for Bacteria and Archaea (Fig. 1), as one would expect, the curve is not asymptotic at all but sharply increasing; prokaryote discovery and understanding is obviously just beginning.

In fact, although many new eukaryote families are named every year, the vast majority of these new names result from advances in phylogenetic understanding, not biological discovery of major new forms of life. The last ten years of Zoological Record suggests that roughly 5–10 truly new families are discovered per year.

In the context of the above question—approximate taxonomic assignment of organisms using DNA sequences—these data suggest that our knowledge of major clades of life is approaching completion. The Global Genome Initiative (GGI; http://ggi.si.edu/) of the Smithsonian Institution via the GGI Knowledge Portal (http://ggi.eol.org/) has tabulated a complete list of families of life, which total 9,650—on the whole a surprisingly small number. 10,000 barcodes, more or less, seems like a feasible goal. If we were able to assemble a complete database of DNA sequences at the family level, would it suffice to identify any eukaryote on Earth to the family level?

While the literature on species identification success of DNA barcodes comprises thousands of studies, only a few have tested their effectiveness at the level of higher taxonomic units. In the seminal paper on DNA barcodes, Hebert et al. (2003) established that animal CO1 sequences can roughly assign taxa to phyla (96% success) or orders (100% success). However, their test was based on a neighbor joining tree-building approach, and it remained unknown if sequence data itself, i.e., percent identity among taxa, can be used in this way. Similarly, Nagy et al. (2012) showed that DNA barcoding in reptiles usually correctly assigned barcodes to species, genus and family. Their approach was phylogenetic: they tested whether including a sequence in tree building rendered the higher group non-monophyletic, which would imply failure. Finally, Wilson et al. (2011) provided a similar tree based test in sphingid moths, and established reliabilities of correct generic and subfamily taxonomic assignments between 74 and 90% using a liberal, and only 66–84% using a strict, tree-based criterion. These authors argued that tree-based methods perform better than sequence comparison methods, but that reliability, of course, depends on the library completeness.

Our project not only contributes original DNA barcode data for Central European spiders, but also works in synergy with the GGI towards a permanent preservation of genomic biodiversity: the formation of a collection of deeply frozen spider tissues and their DNA. We provide: (1) cryo-preserved tissues of reliably identified species of Central European spiders, and their vouchers photographed and deposited in public museums; (2) permanently frozen genomic DNA of these species; (3) publicly accessible DNA barcodes for these species (genetic sequence of cytochrome oxidase I—CO1) as public identification tool (Hebert et al., 2003) to facilitate organism identification, taxonomy, ecology and conservation.

In addition, this project addresses to what extent higher level taxonomic units can be reliably identified using barcodes of unknown spiders, and specifically asks what percent sequence identity in BLAST results is necessary to correctly identify unknown taxa to the Linnaean genus and/or family. Other methods for classification of higher-level taxonomies such as RDP (Wang et al., 2007), UTAX (Edgar, 2010) and MEGAN (Huson et al., 2007) have primarily been developed for studies of microorganisms, using genetic markers for these groups, but less is known about using the CO1 barcoding gene in metazoans. We examine empirical data from Araneae barcode data to ask what is the percent sequence identity value above which 5% or less of higher level (genus/family) taxonomic identifications are incorrect and the extent to which frequency of correct identifications correlated with the number of taxa in this dataset, as would be expected given the dependence of BLAST on the reference database.

## Materials & Methods

### Specimen processing and imaging

We used automated and manual sampling methods for collecting spiders in the field in numerous localities in Slovenia and Switzerland. Faunistic and sampling details are published elsewhere (Čandek et al., 2013; see also 2015 corrigendum). Collected spiders were fixed in absolute ethanol immediately after being caught and the ethanol was replaced on the following day. Spiders were frozen at −80 °C, same day, or as soon as possible. In the laboratory they were identified, labeled, photographed and processed for DNA extraction and sequencing (Čandek et al., 2013; see also 2015 corrigendum). Voucher specimens (voucher codes starting with 0078) are deposited at National Museum of Natural History, Smithsonian Institution (Washington D.C., USA), with duplicates (voucher codes starting with ARA) at Naturhistorisches Museum der Burgergemeinde Bern (Switzerland) and EZ LAB, ZRC SAZU (Ljubljana, Slovenia).

Voucher images are published along with their barcodes (see Table 1) at http://ezlab.zrc-sazu.si/dna. All original sequences generated by this project have been submitted to BOLD systems, and those that BOLD accepted were also submitted to GenBank (Table 1).

### Tissues

After specimen identification and processing, up to four legs (or in the case of very small individuals the whole prosoma) of a spider were removed and stored in fresh absolute ethanol in cryovials. Part of the tissue was used for DNA isolation while the other part remains permanently frozen at −80 °C at GGI facilities. The maintenance and use of these materials abides by the international legal standards and conventions of the biological genetic heritage (The Access and Benefit Sharing agreement as part of the 2010 Nagoya protocol).

**Table 1:** Original sequences this project submitted to BOLD and GenBank (only those on GenBank are also publically available on BOLD, for all others, see http://ezlab.zrc-sazu.si/dna/).

Family	Genus	Species	Sample ID	Process ID	GenBank accession number	Voucher stored at	Collected in
Agelenidae	Agelena	labyrinthica	00786574	SPSLO002-12		MNH, SI	SVN
Agelenidae	Agelena	labyrinthica	ARA0239	SPSLO369-13		EZ LAB	SVN
Agelenidae	Allagelena	gracilens	00786557	SPSLO001-12	KX039062	MNH, SI	SVN
Agelenidae	Coelotes	terrestris	00786563	SPSLO003-12	KX039130	MNH, SI	SVN
Agelenidae	Histopona	torpida	00786599	SPSLO004-12	KX039207	MNH, SI	SVN
Agelenidae	Histopona	torpida	ARA0063	SPSLO339-13	KX039208	EZ LAB	SVN
Agelenidae	Inermocoelotes	anoplus	00786586	SPSLO005-12	KX039220	MNH, SI	SVN
Agelenidae	Inermocoelotes	anoplus	ARA0339	SPSLO392-13	KX039219	EZ LAB	SVN
Agelenidae	Malthonica	silvestris	00786304	SPSLO283-13	KX039239	MNH, SI	SVN
Agelenidae	Malthonica	silvestris	ARA0427	SPSLO468-13	KX039238	EZ LAB	SVN
Agelenidae	Tegenaria	atrica	00786583	SPSLO006-12	KX039170	MNH, SI	SVN
Agelenidae	Tegenaria	atrica	ARA0076	SPSLO341-13	KX039169	EZ LAB	SVN
Amaurobiidae	Amaurobius	erberi	00786571	SPSLO007-12	KX039070	MNH, SI	SVN
Amaurobiidae	Amaurobius	erberi	ARA0120	SPSLO347-13	KX039069	EZ LAB	SVN
Amaurobiidae	Amaurobius	fenestralis	00786389	SPSLO189-12	KX039071	MNH, SI	CHE
Amaurobiidae	Amaurobius	ferox	00786307	SPSLO284-13	KX039072	MNH, SI	SVN
Amaurobiidae	Amaurobius	jugorum	00786585	SPSLO008-12	KX039073	MNH, SI	SVN
Anyphaenidae	Anyphaena	accentuata	00786584	SPSLO009-12	KX039076	MNH, SI	SVN
Anyphaenidae	Anyphaena	sabina	00786551	SPSLO010-12	KX039077	MNH, SI	SVN
Araneidae	Aculepeira	ceropegia	00786570	SPSLO011-12	KX039041	MNH, SI	SVN
Araneidae	Aculepeira	ceropegia	ARA0355	SPSLO405-13	KX039040	NMBE	CHE
Araneidae	Agalenatea	redii	00786368	SPSLO095-12	KX039043	MNH, SI	SVN
Araneidae	Agalenatea	redii	ARA0381	SPSLO429-13	KX039042	EZ LAB	SVN
Araneidae	Araneus	alsine	00786568	SPSLO012-12	KX039079	MNH, SI	SVN
Araneidae	Araneus	angulatus	00786552	SPSLO013-12	KX039081	MNH, SI	SVN
Araneidae	Araneus	angulatus	ARA0001	SPSLO326-13	KX039080	EZ LAB	SVN
Araneidae	Araneus	diadematus	00786593	SPSLO014-12	KX039083	MNH, SI	SVN
Araneidae	Araneus	diadematus	ARA0050	SPSLO336-13	KX039082	EZ LAB	SVN
Araneidae	Araneus	marmoreus	00786575	SPSLO015-12	KX039085	MNH, SI	SVN
Araneidae	Araneus	marmoreus	ARA0030	SPSLO329-13	KX039084	EZ LAB	SVN
Araneidae	Araneus	quadratus	00786572	SPSLO016-12	KX039086	MNH, SI	SVN
Araneidae	Araneus	quadratus	ARA0198	SPSLO362-13	KX039087	NMBE	CHE
Araneidae	Araneus	sturmi	00786561	SPSLO017-12	KX039089	MNH, SI	SVN
Araneidae	Araneus	sturmi	ARA0108	SPSLO345-13	KX039088	EZ LAB	SVN
Araneidae	Araniella	cucurbitina	00786596	SPSLO018-12	KX039090	MNH, SI	SVN
Araneidae	Araniella	opisthographa	ARA0393	SPSLO440-13	KX039091	EZ LAB	SVN
Araneidae	Argiope	bruennichi	00786589	SPSLO019-12	KX039093	MNH, SI	SVN
Araneidae	Argiope	bruennichi	ARA0048	SPSLO335-13	KX039094	EZ LAB	SVN
Araneidae	Cercidia	prominens	00786498	SPSLO021-12	KX039116	MNH, SI	SVN
Araneidae	Cercidia	prominens	00786577	SPSLO020-12	KX039114	MNH, SI	SVN
Araneidae	Cercidia	prominens	ARA0356	SPSLO406-13	KX039115	EZ LAB	SVN
Araneidae	Cyclosa	conica	00786573	SPSLO022-12	KX039135	MNH, SI	SVN
Araneidae	Cyclosa	conica	ARA0380	SPSLO428-13	KX039136	NMBE	CHE
Araneidae	Gibbaranea	bituberculata	00786579	SPSLO023-12	KX039186	MNH, SI	SVN
Araneidae	Gibbaranea	bituberculata	ARA0350	SPSLO400-13	KX039187	EZ LAB	SVN
Araneidae	Hypsosinga	albovittata	00786323	SPSLO191-12	KX039211	MNH, SI	CHE
Araneidae	Hypsosinga	pygmaea	00786555	SPSLO024-12	KX039212	MNH, SI	SVN
Araneidae	Hypsosinga	sanguinea	00786314	SPSLO285-13	KX039213	MNH, SI	SVN
Araneidae	Hypsosinga	sanguinea	ARA0370	SPSLO419-13	KX039214	NMBE	CHE
Araneidae	Larinioides	sclopetarius	00786382	SPSLO096-12	KX039222	MNH, SI	SVN
Araneidae	Leviellus	thorelli	00786591	SPSLO025-12	KX039229	MNH, SI	SVN
Araneidae	Leviellus	thorelli	ARA0353	SPSLO403-13	KX039228	EZ LAB	SVN
Araneidae	Mangora	acalypha	00786590	SPSLO026-12	KX039242	MNH, SI	SVN
Araneidae	Mangora	acalypha	ARA0107	SPSLO344-13	KX039240	EZ LAB	SVN
Araneidae	Mangora	acalypha	ARA0357	SPSLO407-13	KX039241	EZ LAB	SVN
Araneidae	Neoscona	adianta	00786330	SPSLO192-12	KX039282	MNH, SI	SVN
Araneidae	Nuctenea	umbratica	00786594	SPSLO027-12	KX039293	MNH, SI	SVN
Araneidae	Nuctenea	umbratica	ARA0387	SPSLO435-13	KX039292	NMBE	CHE
Araneidae	Parazygiella	montana	00786582	SPSLO028-12	KX039307	MNH, SI	SVN
Araneidae	Parazygiella	montana	ARA0354	SPSLO404-13	KX039308	NMBE	CHE
Araneidae	Singa	nitidula	00786597	SPSLO029-12	KX039376	MNH, SI	SVN
Araneidae	Stroemiellus	stroemi	ARA0169	SPSLO358-13	KX039383	EZ LAB	SVN
Araneidae	Zilla	diodia	00786481	SPSLO097-12	KX039446	MNH, SI	SVN
Araneidae	Zilla	diodia	ARA0342	SPSLO393-13	KX039447	EZ LAB	SVN
Araneidae	Zygiella	x-notata	00786326	SPSLO193-12	KX039450	MNH, SI	CHE
Atypidae	Atypus	piceus	00786580	SPSLO031-12	KX039096	MNH, SI	SVN
Atypidae	Atypus	piceus	ARA0174	SPSLO359-13	KX039097	EZ LAB	SVN
Clubionidae	Clubiona	germanica	00786566	SPSLO032-12	KX039122	MNH, SI	SVN
Clubionidae	Clubiona	kulczynskii	00786404	SPSLO194-12	KX039123	MNH, SI	CHE
Clubionidae	Clubiona	neglecta	00786558	SPSLO033-12	KX039124	MNH, SI	SVN
Clubionidae	Clubiona	pseudoneglecta	00786286	SPSLO286-13	KX039125	MNH, SI	SVN
Clubionidae	Clubiona	reclusa	00786378	SPSLO195-12	KX039126	MNH, SI	CHE
Clubionidae	Clubiona	reclusa	ARA0371	SPSLO420-13	KX039127	NMBE	CHE
Clubionidae	Clubiona	terrestris	00786457	SPSLO098-12	KX039129	MNH, SI	SVN
Clubionidae	Clubiona	terrestris	ARA0242	SPSLO371-13	KX039128	EZ LAB	SVN
Corinnidae	Phrurolithus	minimus	00786559	SPSLO034-12	KX039341	MNH, SI	SVN
Dictynidae	Argenna	subnigra	00786283	SPSLO288-13		MNH, SI	SVN
Dictynidae	Cicurina	cicur	00786548	SPSLO035-12	KX039121	MNH, SI	SVN
Dictynidae	Dictyna	arundinacea	00786369	SPSLO196-12	KX039140	MNH, SI	CHE
Dictynidae	Dictyna	arundinacea	ARA0379	SPSLO427-13	KX039139	NMBE	CHE
Dictynidae	Dictyna	civica	00786511	SPSLO036-12	KX039141	MNH, SI	SVN
Dictynidae	Dictyna	uncinata	00786345	SPSLO197-12	KX039143	MNH, SI	SVN
Dictynidae	Dictyna	uncinata	ARA0423	SPSLO466-13	KX039142	EZ LAB	SVN
Dictynidae	Lathys	humilis	00786473	SPSLO198-12	KX039224	MNH, SI	SVN
Dictynidae	Lathys	humilis	00786550	SPSLO037-12	KX039223	MNH, SI	SVN
Dysderidae	Dasumia	canestrinii	00786581	SPSLO038-12	KX039137	MNH, SI	SVN
Dysderidae	Dysdera	adriatica	00786287	SPSLO289-13	KX039154	MNH, SI	SVN
Dysderidae	Dysdera	adriatica	00786296	SPSLO290-13	KX039155	MNH, SI	SVN
Dysderidae	Dysdera	ninnii	ARA0244	SPSLO373-13	KX039156	EZ LAB	SVN
Filistatidae	Filistata	insidiatrix	00786560	SPSLO040-12	KX039181	MNH, SI	SVN
Filistatidae	Filistata	insidiatrix	ARA0122	SPSLO348-13	KX039182	EZ LAB	SVN
Gnaphosidae	Aphantaulax	cincta	00786470	SPSLO199-12	KX039078	MNH, SI	SVN
Gnaphosidae	Callilepis	schuszteri	00786553	SPSLO041-12	KX039103	MNH, SI	SVN
Gnaphosidae	Callilepis	schuszteri	ARA0333	SPSLO386-13	KX039102	EZ LAB	SVN
Gnaphosidae	Drassodes	lapidosus	00786505	SPSLO099-12	KX039150	MNH, SI	SVN
Gnaphosidae	Drassodes	pubescens	00786273	SPSLO291-13	KX039151	MNH, SI	SVN
Gnaphosidae	Drassyllus	villicus	00786556	SPSLO042-12	KX039153	MNH, SI	SVN
Gnaphosidae	Drassyllus	villicus	ARA0337	SPSLO390-13	KX039152	EZ LAB	SVN
Gnaphosidae	Gnaphosa	bicolor	00786276	SPSLO292-13	KX039188	MNH, SI	SVN
Gnaphosidae	Haplodrassus	silvestris	00786578	SPSLO043-12	KX039196	MNH, SI	SVN
Gnaphosidae	Micaria	aenea	00786384	SPSLO200-12	KX039258	MNH, SI	CHE
Gnaphosidae	Micaria	pulicaria	00786274	SPSLO293-13	KX039259	MNH, SI	SVN
Gnaphosidae	Nomisia	exornata	00786564	SPSLO044-12	KX039291	MNH, SI	SVN
Gnaphosidae	Phaeocedus	braccatus	00786592	SPSLO045-12	KX039330	MNH, SI	SVN
Gnaphosidae	Scotophaeus	scutulatus	00786576	SPSLO046-12	KX039369	MNH, SI	SVN
Gnaphosidae	Scotophaeus	scutulatus	ARA0082	SPSLO343-13	KX039370	EZ LAB	SVN
Gnaphosidae	Trachyzelotes	pedestris	00786279	SPSLO294-13	KX039419	MNH, SI	SVN
Gnaphosidae	Zelotes	apricorum	00786278	SPSLO295-13	KX039441	MNH, SI	SVN
Gnaphosidae	Zelotes	latreillei	00786540	SPSLO047-12	KX039443	MNH, SI	SVN
Gnaphosidae	Zelotes	latreillei	ARA0191	SPSLO360-13	KX039442	EZ LAB	SVN
Gnaphosidae	Zelotes	subterraneus	00786588	SPSLO048-12	KX039445	MNH, SI	CHE
Gnaphosidae	Zelotes	subterraneus	ARA0156	SPSLO355-13	KX039444	NMBE	CHE
Hahniidae	Antistea	elegans	00786405	SPSLO201-12	KX039075	MNH, SI	CHE
Hahniidae	Antistea	elegans	ARA0384	SPSLO432-13	KX039074	NMBE	CHE
Hahniidae	Hahnia	difficilis	00786363	SPSLO202-12	KX039195	MNH, SI	CHE
Hahniidae	Hahnia	difficilis	ARA0399	SPSLO445-13	KX039194	NMBE	CHE
Linyphiidae	Agnyphantes	expunctus	00786328	SPSLO203-12	KX039044	MNH, SI	CHE
Linyphiidae	Agnyphantes	expunctus	ARA0429	SPSLO470-13	KX039045	NMBE	CHE
Linyphiidae	Agyneta	affinis	00786439	SPSLO115-12	KX039049	MNH, SI	CHE
Linyphiidae	Agyneta	affinis	ARA0245	SPSLO374-13	KX039048	NMBE	CHE
Linyphiidae	Agyneta	alpica	00786443	SPSLO116-12	KX039050	MNH, SI	CHE
Linyphiidae	Agyneta	cauta	00786426	SPSLO204-12	KX039052	MNH, SI	CHE
Linyphiidae	Agyneta	cauta	ARA0225	SPSLO367-13	KX039051	NMBE	CHE
Linyphiidae	Agyneta	conigera	00786448	SPSLO100-12	KX039053	MNH, SI	CHE
Linyphiidae	Agyneta	fuscipalpa	00786425	SPSLO218-12		MNH, SI	CHE
Linyphiidae	Agyneta	fuscipalpa	ARA0268	SPSLO378-13		NMBE	CHE
Linyphiidae	Agyneta	gulosa	00786464	SPSLO219-12	KX039054	MNH, SI	CHE
Linyphiidae	Agyneta	innotabilis	00786393	SPSLO220-12	KX039055	MNH, SI	CHE
Linyphiidae	Agyneta	orites	00786419	SPSLO221-12	KX039057	MNH, SI	CHE
Linyphiidae	Agyneta	orites	ARA0403	SPSLO449-13	KX039056	NMBE	CHE
Linyphiidae	Agyneta	rurestris	00786411	SPSLO117-12	KX039058	MNH, SI	CHE
Linyphiidae	Agyneta	rurestris	ARA0419	SPSLO462-13	KX039059	EZ LAB	SVN
Linyphiidae	Agyneta	saxatilis	00786277	SPSLO298-13	KX039060	MNH, SI	SVN
Linyphiidae	Agyneta	simplicitarsis	00786295	SPSLO299-13	KX039061	MNH, SI	SVN
Linyphiidae	Bolyphantes	alticeps	00786465	SPSLO205-12		MNH, SI	CHE
Linyphiidae	Bolyphantes	luteolus	00786397	SPSLO101-12	KX039101	MNH, SI	CHE
Linyphiidae	Bolyphantes	luteolus	ARA0214	SPSLO366-13	KX039100	NMBE	CHE
Linyphiidae	Caracladus	avicula	00786474	SPSLO206-12	KX039104	MNH, SI	CHE
Linyphiidae	Caracladus	avicula	ARA0231	SPSLO368-13	KX039105	NMBE	CHE
Linyphiidae	Caracladus	zamoniensis	00786441	SPSLO102-12	KX039106	MNH, SI	CHE
Linyphiidae	Centromerus	pabulator	00786451	SPSLO207-12	KX039108	MNH, SI	CHE
Linyphiidae	Centromerus	pabulator	ARA0421	SPSLO464-13	KX039107	NMBE	CHE
Linyphiidae	Centromerus	subalpinus	00786412	SPSLO208-12	KX039110	MNH, SI	CHE
Linyphiidae	Centromerus	subalpinus	ARA0250	SPSLO375-13	KX039109	NMBE	CHE
Linyphiidae	Ceratinella	brevipes	00786317	SPSLO234-12	KX039112	MNH, SI	CHE
Linyphiidae	Ceratinella	brevipes	00786450	SPSLO103-12	KX039113	MNH, SI	CHE
Linyphiidae	Ceratinella	brevipes	ARA0363	SPSLO413-13	KX039111	NMBE	CHE
Linyphiidae	Diplocephalus	crassilobus	00786294	SPSLO296-13	KX039144	MNH, SI	SVN
Linyphiidae	Diplocephalus	latifrons	00786461	SPSLO209-12	KX039145	MNH, SI	CHE
Linyphiidae	Diplostyla	concolor	00786533	SPSLO049-12	KX039146	MNH, SI	SVN
Linyphiidae	Drapetisca	socialis	00786587	SPSLO050-12	KX039149	MNH, SI	SVN
Linyphiidae	Drapetisca	socialis	ARA0405	SPSLO451-13	KX039148	EZ LAB	SVN
Linyphiidae	Entelecara	acuminata	00786460	SPSLO210-12	KX039164	MNH, SI	CHE
Linyphiidae	Erigone	atra	ARA0257	SPSLO377-13	KX039171	NMBE	CHE
Linyphiidae	Erigone	dentipalpis	ARA0256	SPSLO376-13	KX039172	NMBE	CHE
Linyphiidae	Erigone	remota	00786416	SPSLO107-12	KX039174	MNH, SI	CHE
Linyphiidae	Erigonella	ignobilis	ARA0164	SPSLO357-13	KX039173	NMBE	CHE
Linyphiidae	Floronia	bucculenta	00786545	SPSLO051-12	KX039183	MNH, SI	SVN
Linyphiidae	Frontinellina	frutetorum	00786567	SPSLO052-12	KX039184	MNH, SI	SVN
Linyphiidae	Frontinellina	frutetorum	ARA0441	SPSLO480-13	KX039185	EZ LAB	SVN
Linyphiidae	Gonatium	hilare	00786565	SPSLO053-12	KX039189	MNH, SI	SVN
Linyphiidae	Gonatium	rubellum	00786318	SPSLO212-12	KX039191	MNH, SI	CHE
Linyphiidae	Gonatium	rubellum	ARA0386	SPSLO434-13	KX039190	NMBE	CHE
Linyphiidae	Gonatium	rubens	00786331	SPSLO213-12	KX039192	MNH, SI	CHE
Linyphiidae	Gonatium	rubens	ARA0358	SPSLO408-13	KX039193	NMBE	CHE
Linyphiidae	Improphantes	nitidus	00786449	SPSLO109-12	KX039218	MNH, SI	CHE
Linyphiidae	Incestophantes	frigidus	ARA0211	SPSLO364-13		NMBE	CHE
Linyphiidae	Kaestneria	dorsalis	00786598	SPSLO054-12	KX039221	MNH, SI	SVN
Linyphiidae	Lepthyphantes	leprosus	00786342	SPSLO214-12	KX039225	MNH, SI	SVN
Linyphiidae	Lepthyphantes	nodifer	ARA0433	SPSLO473-13	KX039226	NMBE	CHE
Linyphiidae	Linyphia	hortensis	00786526	SPSLO112-12	KX039230	MNH, SI	SVN
Linyphiidae	Linyphia	hortensis	ARA0397	SPSLO443-13	KX039231	NMBE	CHE
Linyphiidae	Linyphia	triangularis	00786547	SPSLO056-12	KX039232	MNH, SI	SVN
Linyphiidae	Linyphia	triangularis	ARA0004	SPSLO327-13	KX039233	EZ LAB	SVN
Linyphiidae	Macrargus	rufus	ARA0213	SPSLO365-13	KX039237	NMBE	CHE
Linyphiidae	Mansuphantes	fragilis	00786415	SPSLO114-12	KX039243	MNH, SI	CHE
Linyphiidae	Mansuphantes	fragilis	ARA0276	SPSLO380-13	KX039244	NMBE	CHE
Linyphiidae	Maso	sundevalli	00786400	SPSLO216-12	KX039248	MNH, SI	CHE
Linyphiidae	Maso	sundevalli	ARA0360	SPSLO410-13	KX039247	NMBE	CHE
Linyphiidae	Megalepthyphantes	collinus	00786569	SPSLO057-12	KX039249	MNH, SI	SVN
Linyphiidae	Mermessus	trilobatus	00786395	SPSLO118-12	KX039250	MNH, SI	SVN
Linyphiidae	Metopobactrus	prominulus	00786437	SPSLO119-12	KX039257	MNH, SI	CHE
Linyphiidae	Micrargus	alpinus	ARA0270	SPSLO379-13	KX039260	NMBE	CHE
Linyphiidae	Micrargus	herbigradus	00786466	SPSLO223-12	KX039261	MNH, SI	CHE
Linyphiidae	Microctenonyx	subitaneus	00786463	SPSLO224-12	KX039262	MNH, SI	CHE
Linyphiidae	Microlinyphia	impigra	00786350	SPSLO228-12	KX039263	MNH, SI	CHE
Linyphiidae	Microlinyphia	impigra	ARA0369	SPSLO418-13	KX039264	NMBE	CHE
Linyphiidae	Microlinyphia	pusilla	00786417	SPSLO225-12	KX039265	MNH, SI	CHE
Linyphiidae	Minicia	marginella	00786371	SPSLO120-12	KX039267	MNH, SI	SVN
Linyphiidae	Minicia	marginella	ARA0410	SPSLO455-13	KX039268	NMBE	CHE
Linyphiidae	Minyriolus	pusillus	ARA0285	SPSLO382-13	KX039269	NMBE	CHE
Linyphiidae	Mughiphantes	cornutus	ARA0372	SPSLO421-13	KX039272	NMBE	CHE
Linyphiidae	Mughiphantes	mughi	00786319	SPSLO227-12	KX039274	MNH, SI	CHE
Linyphiidae	Mughiphantes	mughi	00786322	SPSLO217-12	KX039275	MNH, SI	CHE
Linyphiidae	Mughiphantes	mughi	ARA0361	SPSLO411-13	KX039273	NMBE	CHE
Linyphiidae	Mughiphantes	mughi	ARA0411	SPSLO456-13	KX039276	NMBE	CHE
Linyphiidae	Nematogmus	sanguinolentus	00786490	SPSLO162-12	KX039279	MNH, SI	SVN
Linyphiidae	Nematogmus	sanguinolentus	ARA0359	SPSLO409-13	KX039278	NMBE	CHE
Linyphiidae	Neriene	clathrata	ARA0352	SPSLO402-13	KX039287	EZ LAB	SVN
Linyphiidae	Neriene	furtiva	00786471	SPSLO229-12	KX039289	MNH, SI	SVN
Linyphiidae	Neriene	furtiva	ARA0145	SPSLO353-13	KX039288	EZ LAB	SVN
Linyphiidae	Neriene	radiata	ARA0152	SPSLO354-13	KX039290	NMBE	CHE
Linyphiidae	Obscuriphantes	obscurus	00786354	SPSLO231-12	KX039295	MNH, SI	CHE
Linyphiidae	Obscuriphantes	obscurus	ARA0407	SPSLO453-13	KX039294	NMBE	CHE
Linyphiidae	Oedothorax	gibbifer	00786396	SPSLO232-12	KX039296	MNH, SI	CHE
Linyphiidae	Oryphantes	angulatus	ARA0398	SPSLO444-13		NMBE	CHE
Linyphiidae	Ostearius	melanopygius	00786339	SPSLO122-12	KX039297	MNH, SI	SVN
Linyphiidae	Palliduphantes	pallidus	00786341	SPSLO233-12	KX039302	MNH, SI	CHE
Linyphiidae	Panamomops	tauricornis	ARA0375	SPSLO424-13	KX039303	NMBE	CHE
Linyphiidae	Pityohyphantes	phrygianus	00786316	SPSLO236-12	KX039351	MNH, SI	CHE
Linyphiidae	Pityohyphantes	phrygianus	ARA0347	SPSLO397-13	KX039352	NMBE	CHE
Linyphiidae	Pocadicnemis	juncea	00786421	SPSLO237-12	KX039354	MNH, SI	CHE
Linyphiidae	Pocadicnemis	juncea	ARA0409	SPSLO454-13	KX039355	NMBE	CHE
Linyphiidae	Pocadicnemis	pumila	00786422	SPSLO238-12	KX039356	MNH, SI	CHE
Linyphiidae	Porrhomma	pallidum	00786410	SPSLO239-12	KX039357	MNH, SI	CHE
Linyphiidae	Porrhomma	pygmaeum	00786292	SPSLO301-13		MNH, SI	SVN
Linyphiidae	Scotinotylus	alpigena	00786444	SPSLO125-12	KX039367	MNH, SI	CHE
Linyphiidae	Scotinotylus	alpigena	ARA0163	SPSLO356-13	KX039366	NMBE	CHE
Linyphiidae	Scotinotylus	clavatus	00786420	SPSLO240-12	KX039368	MNH, SI	CHE
Linyphiidae	Silometopus	elegans	00786409	SPSLO126-12	KX039373	MNH, SI	CHE
Linyphiidae	Tapinocyba	affinis	00786406	SPSLO127-12	KX039387	MNH, SI	CHE
Linyphiidae	Tapinocyba	affinis	ARA0362	SPSLO412-13	KX039386	NMBE	CHE
Linyphiidae	Tenuiphantes	alacris	00786343	SPSLO241-12	KX039389	MNH, SI	CHE
Linyphiidae	Tenuiphantes	alacris	ARA0420	SPSLO463-13	KX039388	NMBE	CHE
Linyphiidae	Tenuiphantes	cristatus	00786305	SPSLO302-13	KX039390	MNH, SI	CHE
Linyphiidae	Tenuiphantes	cristatus	ARA0418	SPSLO461-13	KX039391	NMBE	CHE
Linyphiidae	Tenuiphantes	flavipes	00786528	SPSLO060-12	KX039392	MNH, SI	SVN
Linyphiidae	Tenuiphantes	flavipes	ARA0336	SPSLO389-13	KX039393	NMBE	CHE
Linyphiidae	Tenuiphantes	jacksoni	00786356	SPSLO242-12		MNH, SI	CHE
Linyphiidae	Tenuiphantes	jacksoni	00786430	SPSLO128-12		MNH, SI	CHE
Linyphiidae	Tenuiphantes	jacksoni	ARA0435	SPSLO475-13		NMBE	CHE
Linyphiidae	Tenuiphantes	jacksonoides	ARA0374	SPSLO423-13	KX039394	NMBE	CHE
Linyphiidae	Tenuiphantes	mengei	00786301	SPSLO300-13	KX039396	MNH, SI	CHE
Linyphiidae	Tenuiphantes	mengei	00786413	SPSLO243-12	KX039397	MNH, SI	CHE
Linyphiidae	Tenuiphantes	mengei	ARA0415	SPSLO459-13	KX039395	NMBE	CHE
Linyphiidae	Tenuiphantes	tenebricola	00786418	SPSLO244-12	KX039398	MNH, SI	CHE
Linyphiidae	Tenuiphantes	tenebricola	ARA0414	SPSLO458-13	KX039399	NMBE	CHE
Linyphiidae	Tenuiphantes	tenuis	00786383	SPSLO129-12		MNH, SI	SVN
Linyphiidae	Tiso	aestivus	ARA0422	SPSLO465-13	KX039413	NMBE	CHE
Linyphiidae	Tiso	vagans	00786351	SPSLO246-12	KX039414	MNH, SI	CHE
Linyphiidae	Tiso	vagans	ARA0401	SPSLO447-13	KX039415	NMBE	CHE
Linyphiidae	Walckenaeria	antica	00786429	SPSLO130-12	KX039421	MNH, SI	CHE
Linyphiidae	Walckenaeria	furcillata	00786431	SPSLO131-12	KX039422	MNH, SI	CHE
Liocranidae	Agroeca	brunnea	00786320	SPSLO247-12	KX039046	MNH, SI	SVN
Liocranidae	Agroeca	brunnea	ARA0392	SPSLO439-13	KX039047	EZ LAB	SVN
Liocranidae	Liocranum	rupicola	00786516	SPSLO061-12	KX039234	MNH, SI	SVN
Liphistiidae	Liphistius	sp	ARA0240	SPSLO482-15	KX039235	EZ LAB	MYS
Lycosidae	Alopecosa	accentuata	00786365	SPSLO248-12		MNH, SI	CHE
Lycosidae	Alopecosa	pulverulenta	00786527	SPSLO063-12	KX039064	MNH, SI	SVN
Lycosidae	Alopecosa	pulverulenta	ARA0349	SPSLO399-13	KX039063	NMBE	CHE
Lycosidae	Alopecosa	sulzeri	00786452	SPSLO249-12	KX039065	MNH, SI	SVN
Lycosidae	Alopecosa	taeniata	00786538	SPSLO062-12	KX039066	MNH, SI	CHE
Lycosidae	Alopecosa	trabalis	00786509	SPSLO064-12	KX039067	MNH, SI	SVN
Lycosidae	Alopecosa	trabalis	ARA0438	SPSLO478-13	KX039068	EZ LAB	SVN
Lycosidae	Arctosa	fulvolineata	00786336	SPSLO250-12		MNH, SI	SVN
Lycosidae	Arctosa	lutetiana	00786407	SPSLO132-12		MNH, SI	SVN
Lycosidae	Arctosa	maculata	00786312	SPSLO305-13	KX039092	MNH, SI	SVN
Lycosidae	Aulonia	albimana	00786524	SPSLO133-12	KX039099	MNH, SI	SVN
Lycosidae	Aulonia	albimana	ARA0338	SPSLO391-13	KX039098	EZ LAB	SVN
Lycosidae	Hogna	radiata	00786502	SPSLO065-12	KX039210	MNH, SI	SVN
Lycosidae	Hogna	radiata	ARA0368	SPSLO417-13	KX039209	EZ LAB	SVN
Lycosidae	Pardosa	agrestis	00786385	SPSLO134-12	KX039309	MNH, SI	SVN
Lycosidae	Pardosa	amentata	00786337	SPSLO251-12	KX039311	MNH, SI	SVN
Lycosidae	Pardosa	amentata	ARA0413	SPSLO457-13	KX039310	NMBE	CHE
Lycosidae	Pardosa	bifasciata	00786453	SPSLO252-12	KX039312	MNH, SI	SVN
Lycosidae	Pardosa	blanda	00786358	SPSLO253-12	KX039314	MNH, SI	CHE
Lycosidae	Pardosa	blanda	ARA0345	SPSLO396-13	KX039313	NMBE	CHE
Lycosidae	Pardosa	cf. lugubris	00786529	SPSLO066-12	KX039316	MNH, SI	CHE
Lycosidae	Pardosa	cf. lugubris	ARA0065	SPSLO340-13	KX039315	EZ LAB	SVN
Lycosidae	Pardosa	ferruginea	00786309	SPSLO306-13	KX039317	MNH, SI	CHE
Lycosidae	Pardosa	hortensis	00786289	SPSLO307-13	KX039318	MNH, SI	SVN
Lycosidae	Pardosa	oreophila	00786310	SPSLO308-13	KX039319	MNH, SI	CHE
Lycosidae	Pardosa	oreophila	00786321	SPSLO254-12	KX039320	MNH, SI	CHE
Lycosidae	Pardosa	oreophila	ARA0348	SPSLO398-13	KX039321	NMBE	CHE
Lycosidae	Pardosa	palustris	00786514	SPSLO067-12	KX039323	MNH, SI	SVN
Lycosidae	Pardosa	palustris	ARA0406	SPSLO452-13	KX039322	NMBE	CHE
Lycosidae	Pardosa	proxima	00786311	SPSLO309-13	KX039324	MNH, SI	SVN
Lycosidae	Pardosa	riparia	00786315	SPSLO310-13	KX039326	MNH, SI	SVN
Lycosidae	Pardosa	riparia	ARA0243	SPSLO372-13	KX039325	NMBE	CHE
Lycosidae	Pirata	piraticus	00786375	SPSLO255-12	KX039346	MNH, SI	CHE
Lycosidae	Pirata	piraticus	ARA0430	SPSLO471-13	KX039347	NMBE	CHE
Lycosidae	Piratula	hygrophila	00786388	SPSLO135-12	KX039348	MNH, SI	SVN
Lycosidae	Piratula	knorri	00786402	SPSLO136-12		MNH, SI	SVN
Lycosidae	Trochosa	spinipalpis	00786344	SPSLO137-12		MNH, SI	SVN
Lycosidae	Trochosa	spinipalpis	ARA0388	SPSLO436-13		EZ LAB	SVN
Lycosidae	Xerolycosa	nemoralis	00786541	SPSLO068-12	KX039424	MNH, SI	CHE
Lycosidae	Xerolycosa	nemoralis	ARA0335	SPSLO388-13	KX039423	NMBE	CHE
Mimetidae	Ero	furcata	00786390	SPSLO256-12	KX039175	MNH, SI	CHE
Miturgidae	Cheiracanthium	erraticum	00786367	SPSLO138-12	KX039117	MNH, SI	SVN
Miturgidae	Cheiracanthium	mildei	00786355	SPSLO139-12	KX039118	MNH, SI	SVN
Miturgidae	Cheiracanthium	punctorium	00786519	SPSLO140-12	KX039120	MNH, SI	SVN
Miturgidae	Cheiracanthium	punctorium	ARA0056	SPSLO337-13	KX039119	EZ LAB	SVN
Nemesiidae	Nemesia	pannonica	00786333	SPSLO311-13	KX039280	MNH, SI	SVN
Philodromidae	Philodromus	albidus	00786272	SPSLO312-13	KX039332	MNH, SI	SVN
Philodromidae	Philodromus	aureolus	00786539	SPSLO069-12	KX039333	MNH, SI	SVN
Philodromidae	Philodromus	cespitum	00786513	SPSLO070-12	KX039335	MNH, SI	CHE
Philodromidae	Philodromus	cespitum	ARA0400	SPSLO446-13	KX039334	EZ LAB	SVN
Philodromidae	Philodromus	dispar	00786492	SPSLO142-12	KX039336	MNH, SI	SVN
Philodromidae	Philodromus	praedatus	00786500	SPSLO071-12	KX039338	MNH, SI	SVN
Philodromidae	Philodromus	praedatus	ARA0404	SPSLO450-13	KX039337	NMBE	CHE
Philodromidae	Philodromus	pulchellus	00786475	SPSLO072-12	KX039339	MNH, SI	SVN
Philodromidae	Philodromus	pulchellus	ARA0344	SPSLO395-13	KX039340	EZ LAB	SVN
Philodromidae	Philodromus	vagulus	00786366	SPSLO257-12		MNH, SI	CHE
Philodromidae	Philodromus	vagulus	ARA0351	SPSLO401-13		NMBE	CHE
Philodromidae	Thanatus	formicinus	00786530	SPSLO073-12	KX039403	MNH, SI	SVN
Philodromidae	Tibellus	macellus	00786493	SPSLO074-12	KX039412	MNH, SI	SVN
Philodromidae	Tibellus	macellus	ARA0334	SPSLO387-13	KX039411	EZ LAB	SVN
Pholcidae	Psilochorus	simoni	00786501	SPSLO076-12	KX039359	MNH, SI	SVN
Pisauridae	Pisaura	mirabilis	00786487	SPSLO144-12	KX039349	MNH, SI	SVN
Pisauridae	Pisaura	mirabilis	ARA0383	SPSLO431-13	KX039350	NMBE	CHE
Salticidae	Evarcha	arcuata	00786332	SPSLO259-12	KX039177	MNH, SI	CHE
Salticidae	Evarcha	arcuata	ARA0062	SPSLO338-13	KX039178	EZ LAB	SVN
Salticidae	Evarcha	falcata	00786408	SPSLO145-12		MNH, SI	SVN
Salticidae	Evarcha	falcata	ARA0037	SPSLO331-13	KX039179	EZ LAB	SVN
Salticidae	Evarcha	jucunda	00786503	SPSLO077-12	KX039180	MNH, SI	SVN
Salticidae	Evarcha	michailovi	00786313	SPSLO313-13		MNH, SI	SVN
Salticidae	Evarcha	michailovi	00786458	SPSLO260-12		MNH, SI	SVN
Salticidae	Evarcha	michailovi	ARA0436	SPSLO476-13		EZ LAB	SVN
Salticidae	Hasarius	adansoni	00786348	SPSLO261-12	KX039197	MNH, SI	SVN
Salticidae	Heliophanus	aeneus	00786293	SPSLO314-13		MNH, SI	SVN
Salticidae	Heliophanus	auratus	00786282	SPSLO315-13	KX039198	MNH, SI	SVN
Salticidae	Heliophanus	cupreus	00786518	SPSLO146-12	KX039199	MNH, SI	SVN
Salticidae	Heliophanus	cupreus	ARA0382	SPSLO430-13	KX039200	NMBE	CHE
Salticidae	Heliophanus	flavipes	00786510	SPSLO147-12	KX039202	MNH, SI	SVN
Salticidae	Heliophanus	flavipes	ARA0396	SPSLO442-13	KX039201	EZ LAB	SVN
Salticidae	Heliophanus	kochii	00786495	SPSLO078-12	KX039203	MNH, SI	SVN
Salticidae	Icius	subinermis	00786381	SPSLO148-12	KX039217	MNH, SI	SVN
Salticidae	Leptorchestes	berolinensis	00786512	SPSLO079-12	KX039227	MNH, SI	SVN
Salticidae	Macaroeris	nidicolens	00786338	SPSLO262-12	KX039236	MNH, SI	SVN
Salticidae	Marpissa	muscosa	00786523	SPSLO080-12	KX039245	MNH, SI	SVN
Salticidae	Marpissa	nivoyi	00786496	SPSLO081-12	KX039246	MNH, SI	SVN
Salticidae	Myrmarachne	formicaria	00786432	SPSLO149-12	KX039277	MNH, SI	SVN
Salticidae	Neon	reticulatus	00786370	SPSLO150-12	KX039281	MNH, SI	SVN
Salticidae	Pellenes	seriatus	00786462	SPSLO263-12	KX039329	MNH, SI	SVN
Salticidae	Pellenes	seriatus	00786504	SPSLO082-12	KX039327	MNH, SI	SVN
Salticidae	Pellenes	seriatus	ARA0439	SPSLO479-13	KX039328	EZ LAB	SVN
Salticidae	Philaeus	chrysops	00786472	SPSLO264-12	KX039331	MNH, SI	SVN
Salticidae	Pseudeuophrys	lanigera	00786280	SPSLO316-13	KX039358	MNH, SI	SVN
Salticidae	Saitis	barbipes	00786507	SPSLO083-12	KX039363	MNH, SI	SVN
Salticidae	Salticus	scenicus	00786362	SPSLO265-12	KX039364	MNH, SI	CHE
Salticidae	Sibianor	aurocinctus	00786377	SPSLO266-12		MNH, SI	CHE
Salticidae	Sibianor	aurocinctus	ARA0385	SPSLO433-13		NMBE	CHE
Salticidae	Sitticus	rupicola	00786525	SPSLO084-12	KX039377	MNH, SI	CHE
Salticidae	Sitticus	rupicola	ARA0378	SPSLO426-13	KX039378	NMBE	CHE
Scytodidae	Scytodes	thoracica	00786521	SPSLO085-12	KX039371	MNH, SI	SVN
Segestriidae	Segestria	senoculata	00786281	SPSLO317-13	KX039372	MNH, SI	SVN
Sparassidae	Micrommata	virescens	00786497	SPSLO086-12		MNH, SI	SVN
Sparassidae	Micrommata	virescens	ARA0365	SPSLO414-13	KX039266	NMBE	CHE
Tetragnathidae	Metellina	mengei	00786536	SPSLO087-12	KX039251	MNH, SI	CHE
Tetragnathidae	Metellina	mengei	ARA0373	SPSLO422-13	KX039252	NMBE	CHE
Tetragnathidae	Metellina	merianae	00786298	SPSLO318-13	KX039253	MNH, SI	CHE
Tetragnathidae	Metellina	merianae	ARA0394	SPSLO441-13	KX039254	EZ LAB	SVN
Tetragnathidae	Metellina	segmentata	00786357	SPSLO152-12	KX039255	MNH, SI	SVN
Tetragnathidae	Metellina	segmentata	ARA0431	SPSLO472-13	KX039256	EZ LAB	SVN
Tetragnathidae	Pachygnatha	degeeri	00786399	SPSLO153-12	KX039300	MNH, SI	SVN
Tetragnathidae	Tetragnatha	nigrita	00786534	SPSLO088-12	KX039400	MNH, SI	SVN
Tetragnathidae	Tetragnatha	nigrita	ARA0041	SPSLO332-13	KX039401	EZ LAB	SVN
Tetragnathidae	Tetragnatha	pinicola	00786361	SPSLO267-12	KX039402	MNH, SI	CHE
Tetragnathidae	Tetragnatha	pinicola	00786520	SPSLO155-12		MNH, SI	SVN
Theridiidae	Asagena	phalerata	00786346	SPSLO156-12	KX039095	MNH, SI	SVN
Theridiidae	Crustulina	guttata	00786454	SPSLO268-12	KX039132	MNH, SI	SVN
Theridiidae	Crustulina	guttata	ARA0437	SPSLO477-13	KX039131	EZ LAB	SVN
Theridiidae	Crustulina	scabripes	00786479	SPSLO089-12	KX039134	MNH, SI	SVN
Theridiidae	Crustulina	scabripes	ARA0137	SPSLO352-13	KX039133	EZ LAB	SVN
Theridiidae	Dipoena	melanogaster	00786506	SPSLO090-12	KX039147	MNH, SI	SVN
Theridiidae	Enoplognatha	afrodite	00786532	SPSLO157-12	KX039160	MNH, SI	SVN
Theridiidae	Enoplognatha	afrodite	ARA0135	SPSLO350-13	KX039159	EZ LAB	SVN
Theridiidae	Enoplognatha	latimana	00786329	SPSLO269-12	KX039161	MNH, SI	CHE
Theridiidae	Enoplognatha	ovata	00786515	SPSLO158-12	KX039163	MNH, SI	SVN
Theridiidae	Enoplognatha	ovata	ARA0367	SPSLO416-13	KX039162	NMBE	CHE
Theridiidae	Episinus	angulatus	00786386	SPSLO159-12	KX039165	MNH, SI	SVN
Theridiidae	Episinus	maculipes	00786488	SPSLO160-12	KX039166	MNH, SI	SVN
Theridiidae	Episinus	truncatus	00786327	SPSLO270-12	KX039168	MNH, SI	CHE
Theridiidae	Episinus	truncatus	ARA0132	SPSLO349-13	KX039167	EZ LAB	SVN
Theridiidae	Euryopis	flavomaculata	00786468	SPSLO271-12	KX039176	MNH, SI	SVN
Theridiidae	Heterotheridion	nigrovariegatum	00786482	SPSLO161-12	KX039206	MNH, SI	SVN
Theridiidae	Heterotheridion	nigrovariegatum	ARA0343	SPSLO394-13	KX039205	EZ LAB	SVN
Theridiidae	Neottiura	bimaculata	00786445	SPSLO163-12	KX039284	MNH, SI	SVN
Theridiidae	Neottiura	bimaculata	ARA0366	SPSLO415-13	KX039283	NMBE	CHE
Theridiidae	Neottiura	herbigrada	00786467	SPSLO272-12	KX039285	MNH, SI	SVN
Theridiidae	Neottiura	suaveolens	00786427	SPSLO164-12	KX039286	MNH, SI	SVN
Theridiidae	Paidiscura	pallens	00786288	SPSLO319-13	KX039301	MNH, SI	SVN
Theridiidae	Parasteatoda	lunata	00786476	SPSLO165-12	KX039304	MNH, SI	SVN
Theridiidae	Parasteatoda	tepidariorum	00786531	SPSLO091-12	KX039305	MNH, SI	SVN
Theridiidae	Parasteatoda	tepidariorum	ARA0329	SPSLO384-13	KX039306	EZ LAB	SVN
Theridiidae	Phylloneta	impressa	00786401	SPSLO273-12	KX039342	MNH, SI	CHE
Theridiidae	Phylloneta	impressa	ARA0428	SPSLO469-13	KX039343	NMBE	CHE
Theridiidae	Phylloneta	sisyphia	00786364	SPSLO274-12	KX039344	MNH, SI	CHE
Theridiidae	Phylloneta	sisyphia	ARA0416	SPSLO460-13	KX039345	NMBE	CHE
Theridiidae	Platnickina	tincta	00786380	SPSLO167-12	KX039353	MNH, SI	SVN
Theridiidae	Robertus	lividus	ARA0201	SPSLO363-13	KX039360	NMBE	CHE
Theridiidae	Robertus	mediterraneus	00786334	SPSLO275-12		MNH, SI	CHE
Theridiidae	Robertus	mediterraneus	00786433	SPSLO168-12		MNH, SI	CHE
Theridiidae	Robertus	scoticus	00786290	SPSLO320-13		MNH, SI	SVN
Theridiidae	Robertus	truncorum	00786435	SPSLO169-12	KX039361	MNH, SI	CHE
Theridiidae	Robertus	truncorum	ARA0280	SPSLO381-13	KX039362	NMBE	CHE
Theridiidae	Sardinidion	blackwalli	00786271	SPSLO321-13	KX039365	MNH, SI	SVN
Theridiidae	Simitidion	simile	00786549	SPSLO170-12	KX039375	MNH, SI	SVN
Theridiidae	Simitidion	simile	ARA0442	SPSLO481-13	KX039374	EZ LAB	SVN
Theridiidae	Steatoda	bipunctata	00786325	SPSLO276-12	KX039380	MNH, SI	CHE
Theridiidae	Steatoda	bipunctata	ARA0029	SPSLO328-13	KX039379	EZ LAB	SVN
Theridiidae	Steatoda	triangulosa	00786489	SPSLO171-12	KX039382	MNH, SI	SVN
Theridiidae	Steatoda	triangulosa	ARA0046	SPSLO334-13	KX039381	EZ LAB	SVN
Theridiidae	Theridion	betteni	00786340	SPSLO277-12	KX039404	MNH, SI	CHE
Theridiidae	Theridion	pinastri	00786480	SPSLO172-12	KX039406	MNH, SI	SVN
Theridiidae	Theridion	pinastri	ARA0136	SPSLO351-13	KX039405	EZ LAB	SVN
Theridiidae	Theridion	varians	00786374	SPSLO173-12	KX039408	MNH, SI	SVN
Theridiidae	Theridion	varians	ARA0043	SPSLO333-13	KX039407	EZ LAB	SVN
Thomisidae	Diaea	livens	00786359	SPSLO174-12	KX039138	MNH, SI	SVN
Thomisidae	Ebrechtella	tricuspidata	00786508	SPSLO092-12	KX039157	MNH, SI	SVN
Thomisidae	Ebrechtella	tricuspidata	ARA0033	SPSLO330-13	KX039158	EZ LAB	SVN
Thomisidae	Heriaeus	hirtus	00786469	SPSLO278-12	KX039204	MNH, SI	SVN
Thomisidae	Misumena	vatia	00786387	SPSLO175-12	KX039270	MNH, SI	SVN
Thomisidae	Misumena	vatia	ARA0081	SPSLO342-13	KX039271	EZ LAB	SVN
Thomisidae	Ozyptila	atomaria	00786522	SPSLO176-12	KX039298	MNH, SI	CHE
Thomisidae	Ozyptila	nigrita	00786499	SPSLO093-12	KX039299	MNH, SI	SVN
Thomisidae	Synema	globosum	00786485	SPSLO177-12	KX039384	MNH, SI	SVN
Thomisidae	Synema	globosum	ARA0390	SPSLO438-13	KX039385	NMBE	CHE
Thomisidae	Thomisus	onustus	00786455	SPSLO280-12	KX039410	MNH, SI	SVN
Thomisidae	Thomisus	onustus	ARA0426	SPSLO467-13	KX039409	EZ LAB	SVN
Thomisidae	Tmarus	piger	00786484	SPSLO178-12	KX039417	MNH, SI	SVN
Thomisidae	Tmarus	piger	ARA0376	SPSLO425-13	KX039418	EZ LAB	SVN
Thomisidae	Xysticus	acerbus	00786483	SPSLO179-12	KX039425	MNH, SI	SVN
Thomisidae	Xysticus	audax	00786347	SPSLO180-12	KX039427	MNH, SI	SVN
Thomisidae	Xysticus	audax	ARA0402	SPSLO448-13	KX039426	EZ LAB	SVN
Thomisidae	Xysticus	bifasciatus	00786543	SPSLO181-12	KX039428	MNH, SI	SVN
Thomisidae	Xysticus	cristatus	00786537	SPSLO182-12	KX039430	MNH, SI	SVN
Thomisidae	Xysticus	cristatus	ARA0389	SPSLO437-13	KX039429	NMBE	CHE
Thomisidae	Xysticus	desidiosus	00786372	SPSLO183-12		MNH, SI	SVN
Thomisidae	Xysticus	erraticus	00786275	SPSLO322-13	KX039431	MNH, SI	SVN
Thomisidae	Xysticus	kempeleni	00786486	SPSLO184-12	KX039432	MNH, SI	SVN
Thomisidae	Xysticus	kochi	00786303	SPSLO323-13	KX039433	MNH, SI	SVN
Thomisidae	Xysticus	kochi	ARA0434	SPSLO474-13	KX039434	EZ LAB	SVN
Thomisidae	Xysticus	lanio	00786477	SPSLO185-12	KX039435	MNH, SI	SVN
Thomisidae	Xysticus	lineatus	00786535	SPSLO186-12	KX039437	MNH, SI	SVN
Thomisidae	Xysticus	lineatus	ARA0304	SPSLO383-13	KX039436	EZ LAB	SVN
Thomisidae	Xysticus	macedonicus	00786376	SPSLO281-12	KX039438	MNH, SI	CHE
Thomisidae	Xysticus	tenebrosus	00786478	SPSLO187-12	KX039440	MNH, SI	SVN
Thomisidae	Xysticus	tenebrosus	ARA0332	SPSLO385-13	KX039439	EZ LAB	SVN
Titanoecidae	Titanoeca	tristis	00786297	SPSLO324-13	KX039416	MNH, SI	SVN
Uloboridae	Hyptiotes	paradoxus	00786546	SPSLO188-12	KX039216	MNH, SI	SVN
Uloboridae	Hyptiotes	paradoxus	ARA0241	SPSLO370-13	KX039215	EZ LAB	SVN
Uloboridae	Uloborus	walckenaerius	00786324	SPSLO282-12	KX039420	MNH, SI	SVN
Zoridae	Zora	spinimana	00786494	SPSLO094-12	KX039449	MNH, SI	SVN
Zoridae	Zora	spinimana	ARA0192	SPSLO361-13	KX039448	NMBE	CHE

**Notes.**

MNH, SI National Museum of Natural History, Smithsonian Institution EZ LAB Evolutionary Zoology Lab ZRC SAZU; NMBE Naturhistorisches Museum der Burgergemeinde Bern SVN Slovenia CHE Switzerland MYS Malaysia

### Molecular procedures

At Laboratories of Analytical Biology (National Museum of Natural History, Smithsonian Institution, hereafter LAB), specimens were extracted using the AutoGenPrep phenol-chloroform automated extractor (AutoGen). Samples were digested overnight in buffer containing proteinase-k before extraction. At EZ Lab, specimens were extracted using the Mag MAX™ Express magnetic particle processor Type 700 with DNA Multisample kit (Applied Biosystems, Foster City, CA, USA) following the manufacturer’s protocols with modifications (Vidergar, Toplak & Kuntner, 2014).

At EZ Lab PCR was carried out using mainly primers LCO1490 and HCO2198 (Folmer et al., 1994). Standard reaction volume was 35 µL containing 2.3 mM MgCl_2_ (Promega), 0.15 mM each dNTP (Biotools), 0.4 µM of each primer, 0.2 µL 10 mg/mL BSA (Promega), 0.2 µL GoTaqFlexi polymerase (Promega) and 2 µL DNA. PCR cycling conditions were as follows: an initial denaturation step of 2 min at 94 °C followed by 35 cycles of 40 s at 94°C, 1 min at 48 °–52 °C, 1 min at 72 °C, with final extension at 72 °C for 3 min. Additional primers were used for PCR for a few problematic specimens: dgLCO1490 and dgHCO2198 (Meyer & Paulay, 2005) and the reverse primer Chelicerate-R2 (Barrett & Hebert, 2005). Cycling parameters for difficult specimens were: 20 cycles of usual cycling protocol (above) followed by 15 cycles of 1.5 min at 94 °C, 1.5 min at 52 °C and 2 min at 72 °Cm version 5.6.6 (Kearse et al., 2012). EZ Lab PCR products were sent to be Sanger sequenced at Macrogen Inc. (Amsterdam, Netherlands), and the sequences were aligned, checked for sequencing errors and trimmed to match the barcode region in Geneious Pro version 5.6.6 (Kearse et al., 2012).

At LAB, PCR was carried out using the primer pair LCO1490 (Folmer et al., 1994) and Chelicerate-R2 (Barrett & Hebert, 2005). A 10 µL reaction mix contained 2.5 mM MgCl_2_ 0.3 µM of each primer, 0.5 mM dNTPs, and 5 units of Biolase DNA polymerase (Bioline). PCR cycling conditions were as follows: 35 cycles of 30 s at 95 °C, 30 s at 48 °C, 45 s at 72 °C. PCR products were cleaned with ExoSAP-IT (Affymetrix), Sanger sequenced using Big Dyes (Life Technologies) and run on a 3730xl DNA sequencer (Applied Biosystems). Sequences were examined for quality and trimmed to the standard barcode segment (649 bp) using Sequencher 5.01 (Gene Codes).

### Barcode library

While we targeted 649 bp long DNA barcodes we also submitted (Table 1) 18 shorter fragments (>570 bp) that still satisfy the requirements of The Barcode of Life Data System BOLD systems (Ratnasingham & Hebert, 2007). We combined the 297 species barcodes from this study with publically available Araneae sequences from BOLD retrieved 4 December 2013, for a total of 816 species sequences, which formed the test library for this study. Sequences from BOLD were initially included if the sequence length was at least 600 bases and identification was to species. We further filtered and curated the data to exclude sequences whose identification was anonymous or by non-arachnologists, diverged dramatically from all other spider sequences, or for other reasons the sequences were not deemed to be reliable. After having discarded the above, we did not assess the accuracy of every remaining sequence, as it is well known that both BOLD and GenBank contain errors of various kinds, and we wanted our test library to reflect real world conditions. A single sequence was chosen per species from BOLD using these criteria and added to the original sequences from this project, resulting in 816 species representing 313 genera and 49 families (Table 1 and Table S2). Eighteen sequences were singletons at the family level; the maximum number of species per family was 224. 157 sequences were singletons at the genus level; the maximum number of species per genus was 34.

The standalone BLAST+ suite 2.2.28 (Altschul et al., 1990; Zhang et al., 2000) was used to create a custom BLAST database from these sequences. Each sequence was then queried against the full set using blastn (MegaBLAST task, minimum e value of 1e–10, maximum of top ten hits other than the hit of the query to itself). For each hit the percent of identical nucleotides in the aligned region (PIdent) was calculated by BLAST. An advantage of using BLAST is the local nature of the alignment hits returned. This will account for differences in sequence lengths in the dataset, which may otherwise affect pairwise identity calculations of complete alignments. A possible outcome of BLAST results are short aligned regions that have high similarity but omit much of the queried sequence. To investigate this, we compared lengths of aligned regions with query sequence lengths to determine the prevalence of this in this dataset. Custom Python scripts (GitHub https://github.com/mkweskin/spider-blast) were used to parse the results, removing the match of the query to itself and to score whether hits matched the genus and family of the query sequence or not. Obviously, if the generic identification matched, the family identification also matched; families therefore always match more often than genera.

On the other hand, singleton generic sequences cannot match correctly at the genus level (for spiders or other poorly known diverse groups), and, likewise, singleton family sequences cannot match correctly at the family level (for spiders or other poorly known diverse groups). We included singletons as targets in order to model more realistically BLAST searches against the BOLD database (many sequences in BOLD are higher level singletons), and also to test more strongly the ability of sequences with two or more species per either genus or family to match correctly. Including 18 singleton family sequences and 157 singleton genus sequences, therefore, increases the probability of misidentification at either ranks and more strongly tests the usefulness of barcodes as supraspecific identification tools.

However, because the 18 unique family sequences must fail at both the family and genus levels, and the 157 unique genus level sequences must fail at the genus level, these necessary failures were not included in the overall assessments of the ability of barcode sequences to provide accurate identifications at supraspecific levels.

## Results

**Figure 2:**
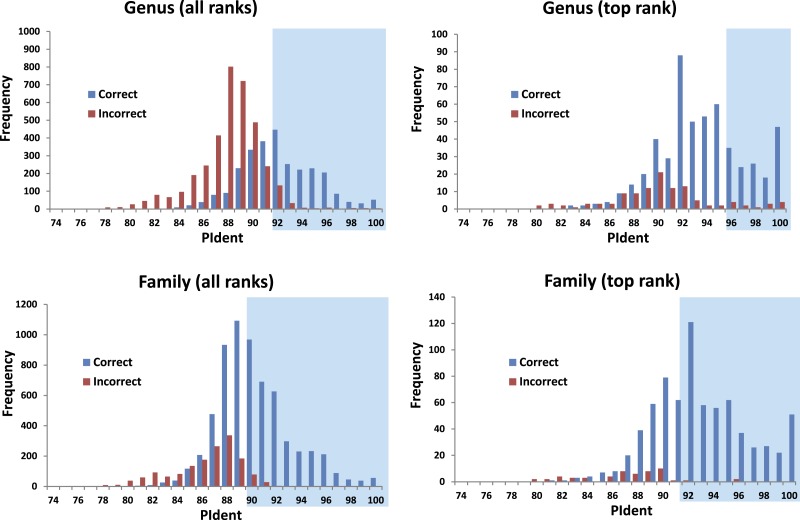
Results from the barcode matching test. Frequency distributions of correct and incorrect identifications by percent sequence identity (PIdent) for the top ten and/or best hits at the genus and family level. Shaded areas include hits where no more than 5% of identifications were incorrect.

The 816 query sequences returned 8,159 total hits with one query only returning nine hits and all others ten (Table S1). PIdent scores ranged from 75% to 100%. We also examined the length of the sequence matched compared to the entire sequence length. 8,114 hits (>99%) matched to 90% or more of the query sequence length indicating that these results represent matches to large portions of the query validating the use of Percent Sequence Identity in the BLAST hits rather than computing the value for a global alignment between sequences. Figure 2 shows the frequency distributions of PIdent values of correct and incorrect identifications at the genus and family rank.

**Figure 3:**
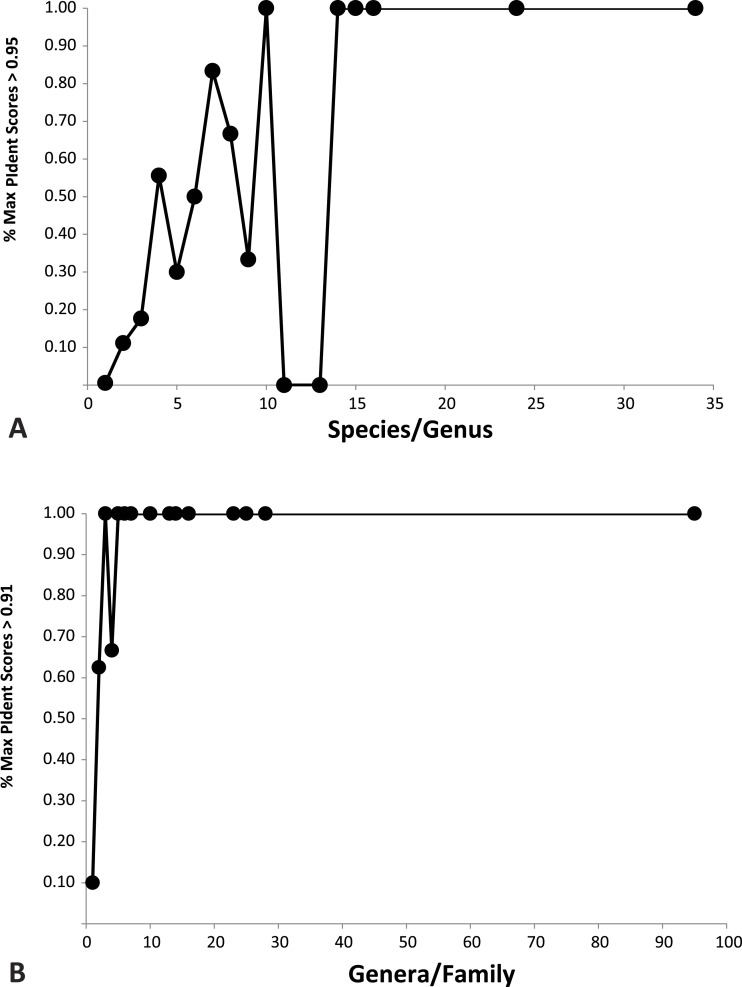
Importance of library representation. Relation between proportion of best sequence identity and numbers of species per genus (A), and genera per family (B). Heuristic thresholds to delimit incorrect from correct identifications were 95 and 91 for genus and family, respectively.

1. 95% of incorrect genus identifications were below PIdent = 95 when all hits for all queries are included, which suggests the latter value as a heuristic threshold to delimit incorrect from correct identifications (for these data). For only the highest rank hits whose PIdent ≥95, 98% of genus identifications were correct.
2. 95% of incorrect family identifications were below PIdent = 91 when all hits for all queries are included, which suggests the latter value as a heuristic threshold to delimit incorrect from correct identifications (for these data). For only the highest rank hits whose PIdent ≥91, 97% of family identifications were correct.
3. Library accuracy is crucial, but sequencing, labelling, and identification errors are difficult to detect *a priori*. The highest ranked incorrect family identification was *Meta menardi* (Tetragnathidae) to *Steatoda grossa* (Theridiidae), at PIdent = 96. Further study of the *M. menardi* sequence shows that the BOLD record is probably a mislabeled *Steatoda*. The first true incorrect family identification occurs at a PIdent value of 88; the best hit for *Octonoba* (Uloboridae) is *Amaurobius* (Amaurobiidae).
4. For the 136 genera with at least two species in the library, 76% (*n* = 103) best matched congeners. Thirty-three failed, perhaps because sequences were incorrectly identified taxonomically, or the sequence itself may be erroneous, or perhaps due to non-monophyly of genera.
5. The distributions of PIdents for correct family and genus identifications differ significantly from the distributions of incorrect identifications (Fig. 2).
6. Plotted against increasing numbers of species/genus, and genera/family, the proportion of top ten PIdent values that exceed the above suggested threshold values increases. Roughly speaking, 15 species per genus, and 5 genera per family, are sufficient to ensure that best hits represent correct identifications (Fig. 3).

## Discussion

We show that standard DNA barcodes can accurately assign unknown specimens to genus and family given sufficient sequence identity and sufficient taxonomic representation in the database. Accurate identification (PIdent above which less than 5% of identifications were incorrect) occurred for genera at PIdent values > 95 and families at PIdent values ≥91, suggesting these as heuristic thresholds for generic and familial identifications in spiders (shaded in Fig. 2). Accuracy of identification increases with numbers of species/genus and genera/family; above five genera per family and 15 species per genus all identifications were correct (Fig. 3).

The accurate identification of specimens remains a critical challenge for megadiverse groups such as arthropods, most other invertebrates, plants, fungi, protists etc. Morphological identification to species, or even more inclusive taxonomic ranks like genera and families, in many cases requires extensive training, and for most groups taxonomic expertise is limited and dwindling—the so called ‘taxonomic impediment’ (Rodman & Cody, 2003; Agnarsson & Kuntner, 2007). DNA barcodes have been proposed as convenient tools to overcome this impediment by making identification a purely technical procedure available to any interested researcher or even ‘citizen scientists.’ However, the accuracy of such a tool strongly depends on the scope and quality of the barcode library (Smit, Reijnen & Stokvis, 2013). Currently available data on databanks like BOLD and GenBank are extensive for some groups, yet the vast majority of species on earth have not yet been barcoded, much less discovered and described taxonomically—each of these tasks is enormous. Even for existing barcoding data, numerous sequences lack accurate taxonomic identification (Collins & Cruickshank, 2013), limiting their utility (e.g., only 58% of Araneae in BOLD are identified to species, and of those many are not correctly identified, as shown in our results; see also Shen, Chen & Murphy, 2013; Blagoev et al., 2016). Therefore, the identification of unknown specimens through blasting against BOLD or GenBank will be inaccurate if the databases lack close hits or contain errors. While the ideal database would allow species-level identification by containing barcodes from expertly identified and vouchered specimens of all species, we hypothesized that rapid surveys of well-known biotas can help quickly to build valuable tools allowing identification of larger clades such as genera and families.

Although we were careful to screen available barcode sequences from BOLD to produce a test library with as few errors as possible, it is certainly possible that errors remained, either due to mistakes in the lab or taxonomic identifications of vouchers. For example, *Meta menardi* (Tetragnathidae) blasted to *Steatoda grossa* (Theridiidae) at PIdent = 96, and BLAST searches on GenBank suggest this *Meta* sequence is actually a *Steatoda*. Likewise, the linyphiids *Agyneta orites* and *Incestophantes frigidus* sequences were identical; one of these records is probably wrong. These sorts of errors bias identifications and limit utility of barcodes. Other examples of identical barcode sequences were all congeners, and therefore are less likely to involve errors but could indicate faults in taxonomy: *Arctosa maculata* and *A. fulvolineata*, *Bolyphantes luteolus* and *B. alticeps*, *Pardosa alacris* and *P. trifrons*, and *Pityohyphantes tacoma* and *P. cristatus*. Likewise, the genus *Neriene* (Linyphiidae) seems non-monophyletic and identifications were thus not accurate.

## Conclusions

These results suggest that accurate assignment of unknown taxa to genus and family is feasible through DNA barcoding. Database quality is crucial. Numbers of potential matches at generic and familial ranks also affect the probability that an unknown sequence will blast best to the correct family or genus. Unlike the inventory of species, biological discovery of family-level clades of life also seems far advanced—few eukaryotic families, apparently, remain to be discovered. Taken together, these results suggest that barcode-targeted sequencing of exemplars from all families of life (and most genera, if possible) should be an important scientific priority. It would enable approximate taxonomic identification of any organism anywhere on Earth by rapid, cheap, purely technical procedures requiring no specialist knowledge—certainly an important milestone in the on-going attempt to discover, classify, and understand the Earth’s biota.

##  Supplemental Information

10.7717/peerj.2201/supp-1 Table S1 The results of the barcode matching test.

10.7717/peerj.2201/supp-2 Table S2 The downloaded sequences used in the species comparison.

10.7717/peerj.2201/supp-3 Table S3 Original sequences this project submitted to BOLD and GenBank (only those on GenBank are also publically available on BOLD, for all others, see http://ezlab.zrc-sazu.si/dna/). Legend: MNH, SI = National Museum of Natural History, Smithsonian Institution; EZ LAB = Evolutionary Zoology Lab, ZRC SAZU; NMBE = Naturhistorisches Museum der Burgergemeinde Bern; SVN = Slovenia; CHE = Switzerland; MYS = Malaysia.

## Additional Information and Declarations

### DNA Deposition

The following information was supplied regarding the deposition of DNA sequences: GenBank accessions are in Table 1 and are also publicly available on BOLD. For all others, see http://ezlab.zrc-sazu.si/dna/.

### Data Availability

The following information was supplied regarding data availability: Github: https://github.com/mkweskin/spider-blast
